# Supplementary material for: Structural basis for an early stage of the photosystem II repair cycle in Chlamydomonas reinhardtii
Source: Nat Commun. 2024 Jun 18;15:5211. doi: 10.1038/s41467-024-49532-2 (PMC11189392; doi:10.1038/s41467-024-49532-2)
Supplement: Supplementary file 1 — Supplementary Information file [file 41467_2024_49532_MOESM1_ESM.pdf]

Structural basis for an early stage of the photosystem II repair cycle in *Chlamydomonas reinhardtii*

Anjie Li[1,2,*], Tingting You[3,*], Xiaojie Pang[2,4], Yidi Wang[1,2], Lijin Tian[2,4], Xiaobo Li[3,5,†] and Zhenfeng Liu[1,2,†]

[1]Key Laboratory of Biomacromolecules (CAS), National Laboratory of Biomacromolecules, CAS Centre for Excellence in Biomacromolecules, Institute of Biophysics, Chinese Academy of Sciences, Beijing 100101, China.

[2]College of Life Sciences, University of Chinese Academy of Sciences, Beijing 101408, China.

[3]Key Laboratory of Growth Regulation and Translational Research of Zhejiang Province, School of Life Sciences, Westlake University, Hangzhou 310024, China.

[4]Key Laboratory of Photobiology, Institute of Botany, Chinese Academy of Sciences, Beijing 100093, China.

[5]Institute of Biology, Westlake Institute for Advanced Study, Hangzhou 310024, China.

[*]These authors contributed equally.

[†]Correspondence should be addressed to Z.L. (liuzf@ibp.ac.cn) and X.L. (lixiaobo@westlake.edu.cn)

Supplementary Figures 1-12 and Supplementary Tables 1-5

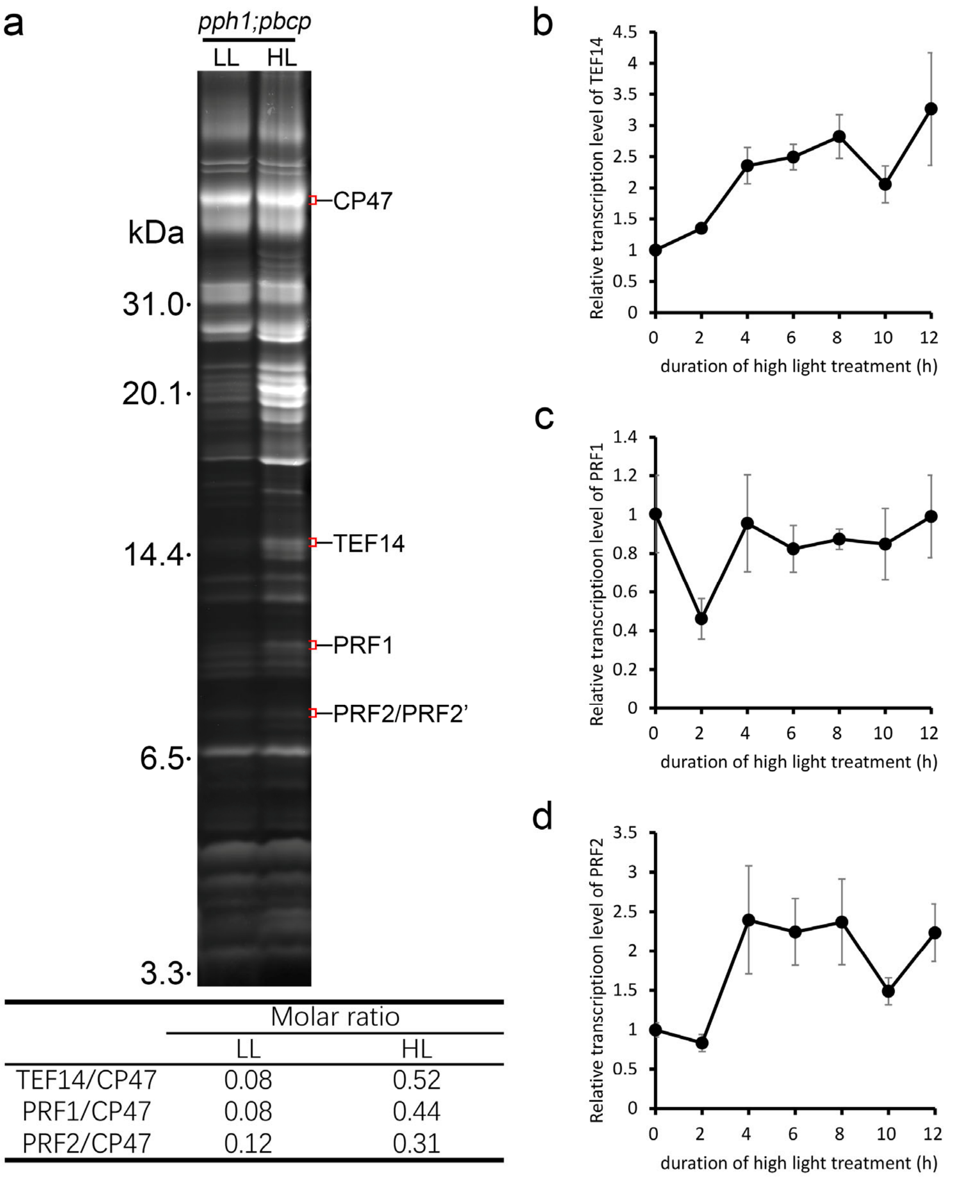

	Molar ratio	
	LL	HL
TEF14/CP47	0.08	0.52
PRF1/CP47	0.08	0.44
PRF2/CP47	0.12	0.31

Supplementary Fig. 1: Quantitative analyses on the relative protein levels and transcription levels of TEF14, PRF1 and PRF2. a, The SDS-PAGE analysis of the PSII core complex samples prepared from the high-light (HL) and low-light (LL) adapted cells, respectively. The gel was stained by the SYPRO Ruby dye and scanned on a Typhoon FLA 9500 imager. The relative amount of protein in the designated band is quantified as the fluorescence intensity of the band divided by the corresponding protein molecular weight. The molar ratios were calculated as the ratios between the relative amounts of the target protein (TEF14, PRF1 or PRF2/PRF2') and that of CP47 band in the same lane of the same gel. The experiment was conducted once. **b-d,** The change of transcript levels of TEF14, PRF1 or PRF2 in response to the high-light treatment. The light intensity for treating the *C. reinhardtii* cells is at 330 μmol photons m^{-2} s^{-1}. The relative transcription levels are presented as mean ± SD of three biological replicates (n = 3, each containing three technical replicates). The transcription level at each time point was normalized to the level measured at 0 hour.

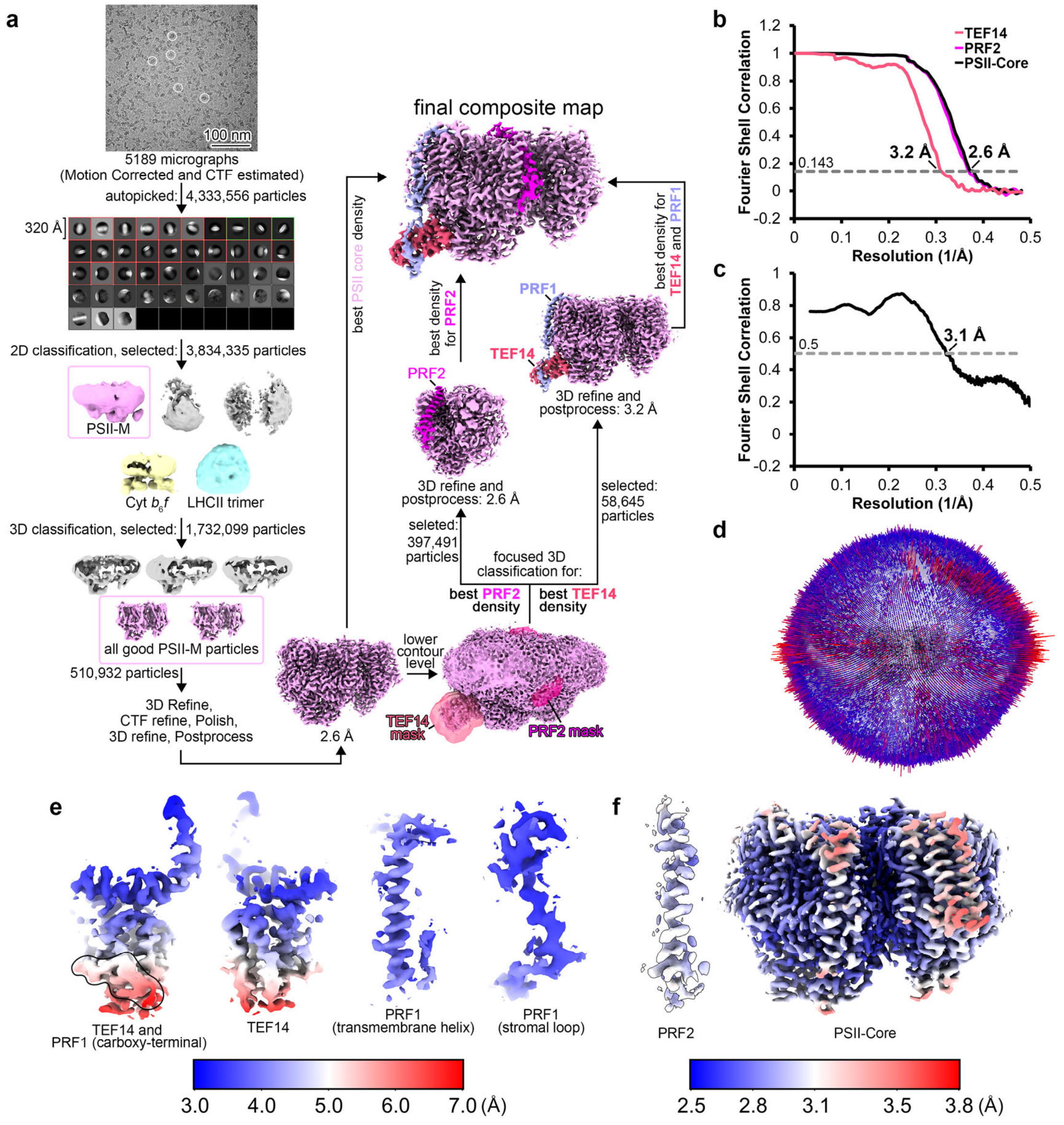

Supplementary Fig. 2: Single particle cryo-EM analysis work flow and evaluation for the PSII-TPP complex from the *C. reinhardtii pph1;pbcp* mutant strain. a, The overall scheme for the single particle cryo-EM data processing procedure. Representative PSII-M particles are highlighted by white circles in the micrograph. The micrograph is a representative of 5189 micrographs with similar quality. All selected classes potentially containing PSII-M particles are framed by rectangles, while the representative PSII-M 2D averaged classes are highlighted by green squares. **b,** The gold standard Fourier shell correlation (GSFSC) curves of local refined density maps with a resolution-cutoff threshold at 0.143. **c,** The Fourier shell correlation (FSC) curves between the structural model and the composite cryo-EM density map of the PSII-TPP complex. **d,** Representative orientational distribution plot of 58,645 3D-refined particles of the PSII-TPP complex with the best TEF14 and PRF1 density at an overall resolution of 3.2 Å. The map with the best densities of the PSII core, TEF14/PRF1 and PRF2 region yielded an E_{od} value of 0.8, 0.8 and 0.79, respectively. The high E_{od} values

indicate that the orientational distributions of the three datasets are all in a reasonable range. **e** and **f,** Local resolution estimations of the TEF14, PRF1, PRF2 and PSII core part of the final composite map. The carboxy-terminal region of PRF1 is circled by the black line in (**e**).

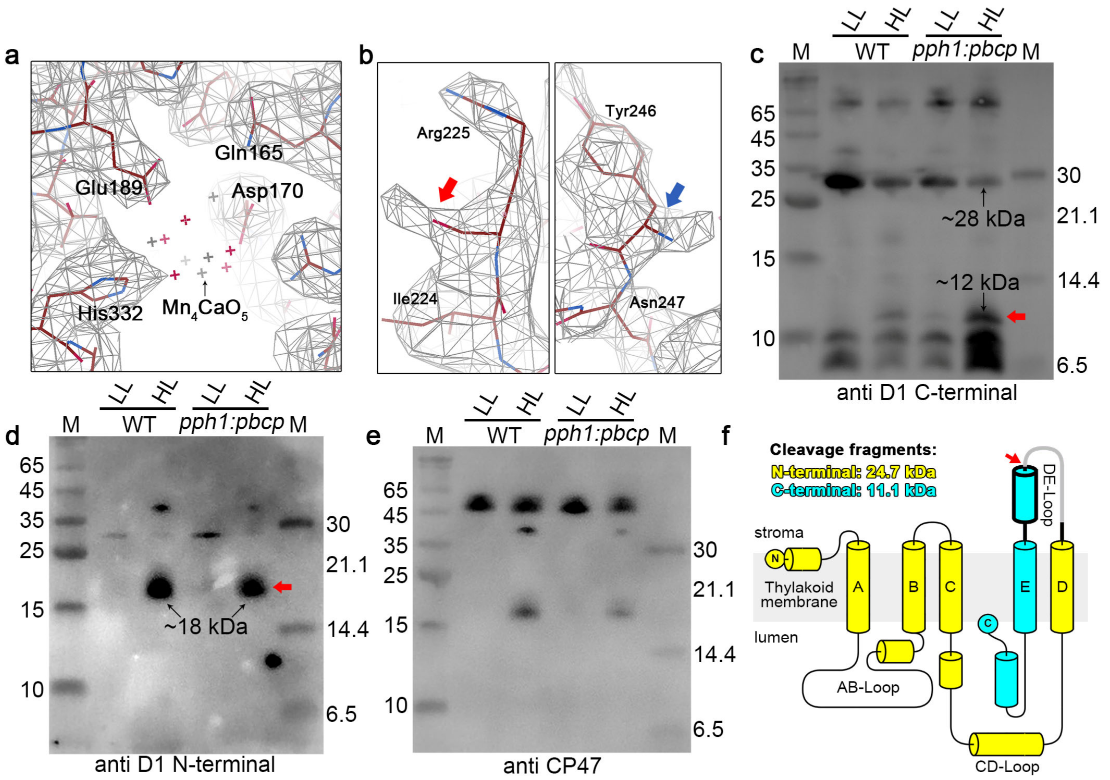

Supplementary Fig. 3: The structural and biochemical evidences suggesting the D1 subunit in the PSII-TPP complex is at a damaged state. a, The cryo-EM density of the local region around the empty Mn-cluster binding site in the PSII-TPP complex. The atomic model of the Mn-cluster (gray and red crosses) is docked into its binding site by fitting the model of a PSII-LHCII supercomplex (PDB ID: 6KAC) into the PSII-TPP density map. **b,** The cryo-EM densities at the local regions around Arg225 and Tyr246 residues of the D1 subunit in the PSII-TPP complex. The α-carboxyl group of Arg225 and the α-amino group of Tyr246 are indicated respectively as red and blue arrows. Evidently, the cryo-EM density for the loop connecting the two residues is either very weak or absent. **c and d,** The western blot analysis of the PSII-M or PSII-TPP samples prepared from the WT and *pph1;pbcp* strains acclimated under low light (LL) and high light (HL) conditions. The antibodies against the carboxy-terminal (**c**) and amino-terminal fragment (**d**) of D1 were used to detect potential degradation products of the D1 proteins. The bands corresponding to the D1 amino/carboxy-terminal fragments are indicated by red arrows. Note that the apparent molecular weight detected for the amino-terminal fragment of D1 in (**d**) appears lower than expected (~25 kDa as estimated in (**f**). The highly hydrophobic nature of the amino-terminal fragment of the D1 apoprotein might make the protein resistant to the denaturing effect of SDS so that it could migrate on the gel in a partially unfolded state and faster than expected during SDS-PAGE. It is also the case for the full length D1 apoprotein which also migrates faster than expected on SDS-PAGE gel. **e,** The western blot of the SDS-PAGE gel probed with the antibody against CP47 as the loading control for the different PSII-M or PSII-TPP complex samples. LL and HL refer to the conditions with light intensities of 20 and 330 μmol·photons $m^2 \cdot s^{-1}$, respectively. The western blots in **c-e** are representatives of two biological replicates with similar results. **f,** A topological

diagram of the D1 apoprotein. The putative proteolytic degradation site at the DE loop is marked by a red arrow. After proteolytic cleavage at the site, the large fragment in yellow corresponds to a 24.7-kDa amino-terminal fragment and the smaller one in cyan is a 11.1-kDa carboxy-terminal fragment. The local region of the DE loop with unobserved cryo-EM density is shown as gray.

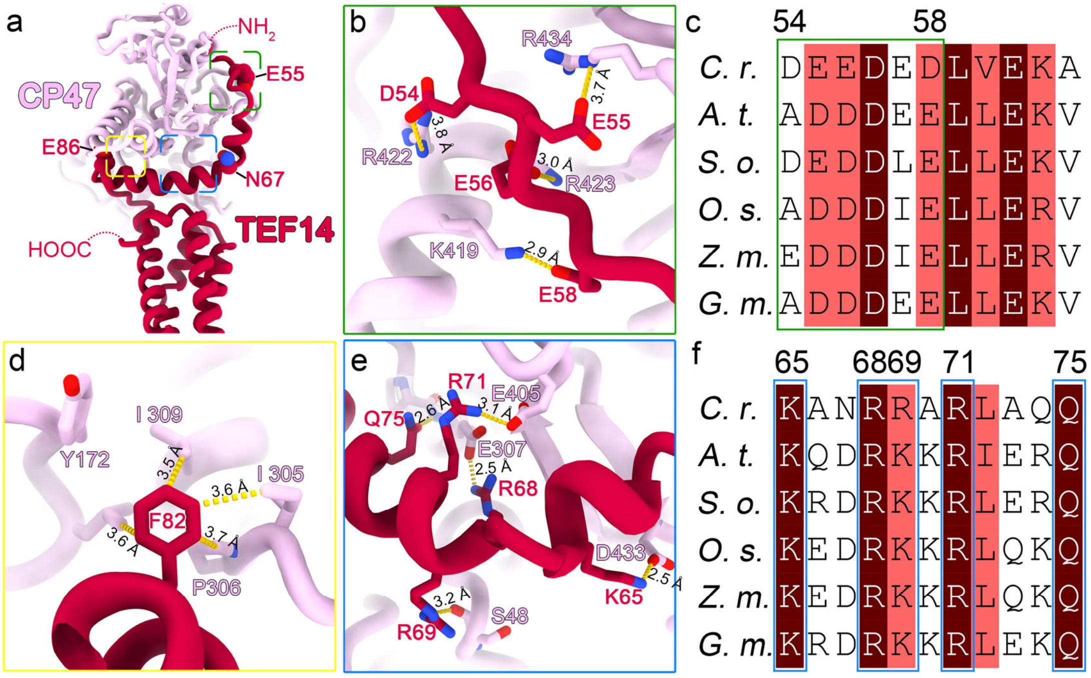

Supplementary Fig. 4: Interactions between TEF14 and CP47. a, The binding site of TEF14 at the lumenal surface of CP47. E55, N67 and E86 from the amino-terminal region of TEF14 are shown as spheres. **b, d** and **e,** The detailed interactions between three local regions at the amino-terminal helix of TEF14 and their nearby amino acid residues from CP47. **c** and **f,** Sequence alignment of *Cr*TEF14 with various MPH2 homologs from higher plants showing the conserved CP47-binding residues (framed by green and blue rectangles corresponding to those shown in **d** and **g** respectively). *A. t.*, *S. o.*, *O. s.*, *Z. m.* and *G. m.* stand for *Arabidopsis thaliana*, *Spinacia oleracea*, *Oryza sativa*, *Zea mays* and *Glycine max*, respectively.

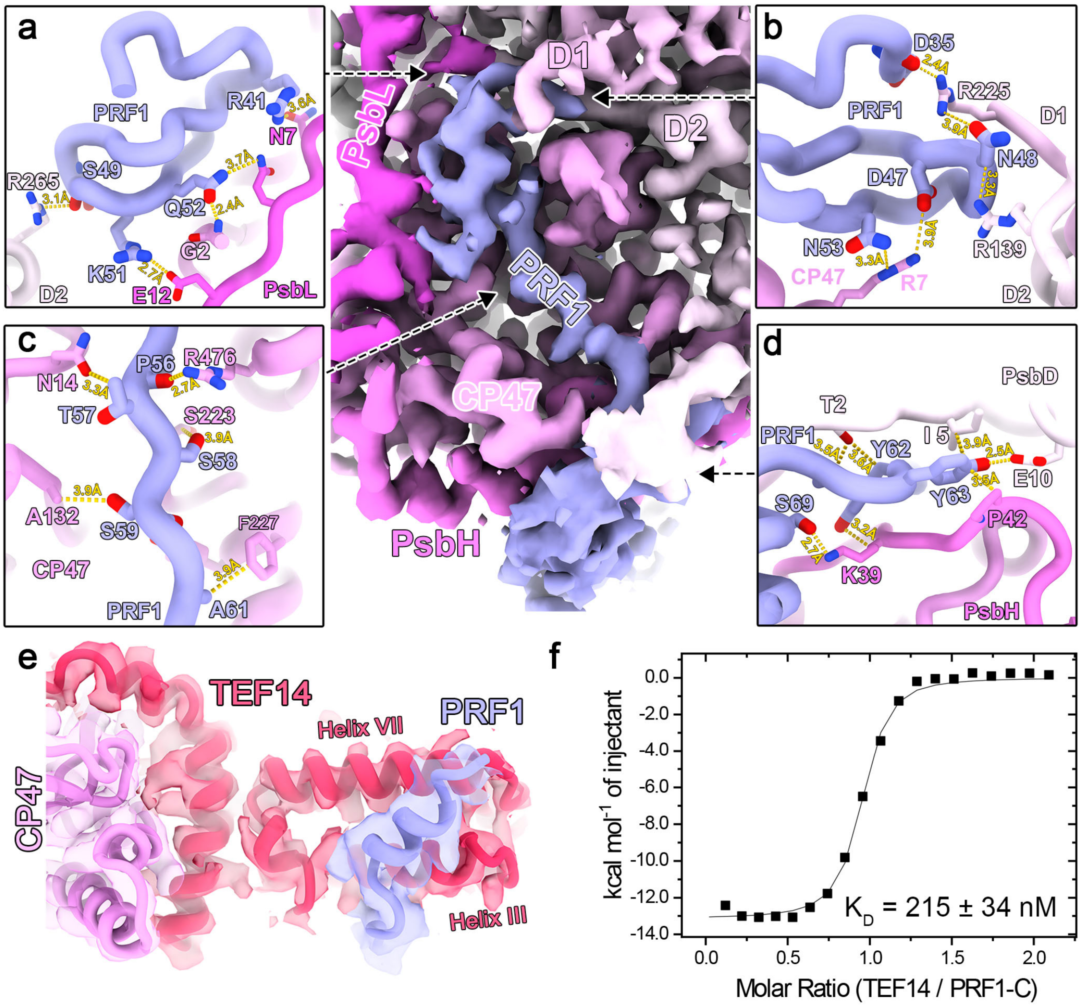

Supplementary Fig. 5: Interactions between PRF1 and PSII-M/TEF14. a and **b,** The amino-terminal hairpin structure of PRF1 forms extensive interactions with amino acid residues from D1, D2, CP47 and PsbL. **c,** The loop connecting the hairpin motif and the transmembrane helix of PRF1 at the amino-terminal region is embedded in a stromal surface groove of CP47. **d,** Two consecutive tyrosine residues from PRF1 are sandwiched between D2 and PsbH. **e,** The interactions between the carboxy-terminal helix of PRF1 and the five-helical-bundle domain of TEF14. The cartoon model is superposed on the Cryo-EM density map (transparent). **f,** The isothermal titration calorimetry (ITC) analysis on the kinetics of interactions between TEF14 and the carboxy-terminal region of PRF1. The result is fitted with the single-site-binding isotherm model with ΔH = -13.1 ± 0.2 kcal/mol and K_D = 215.0 ± 34.2 µM.

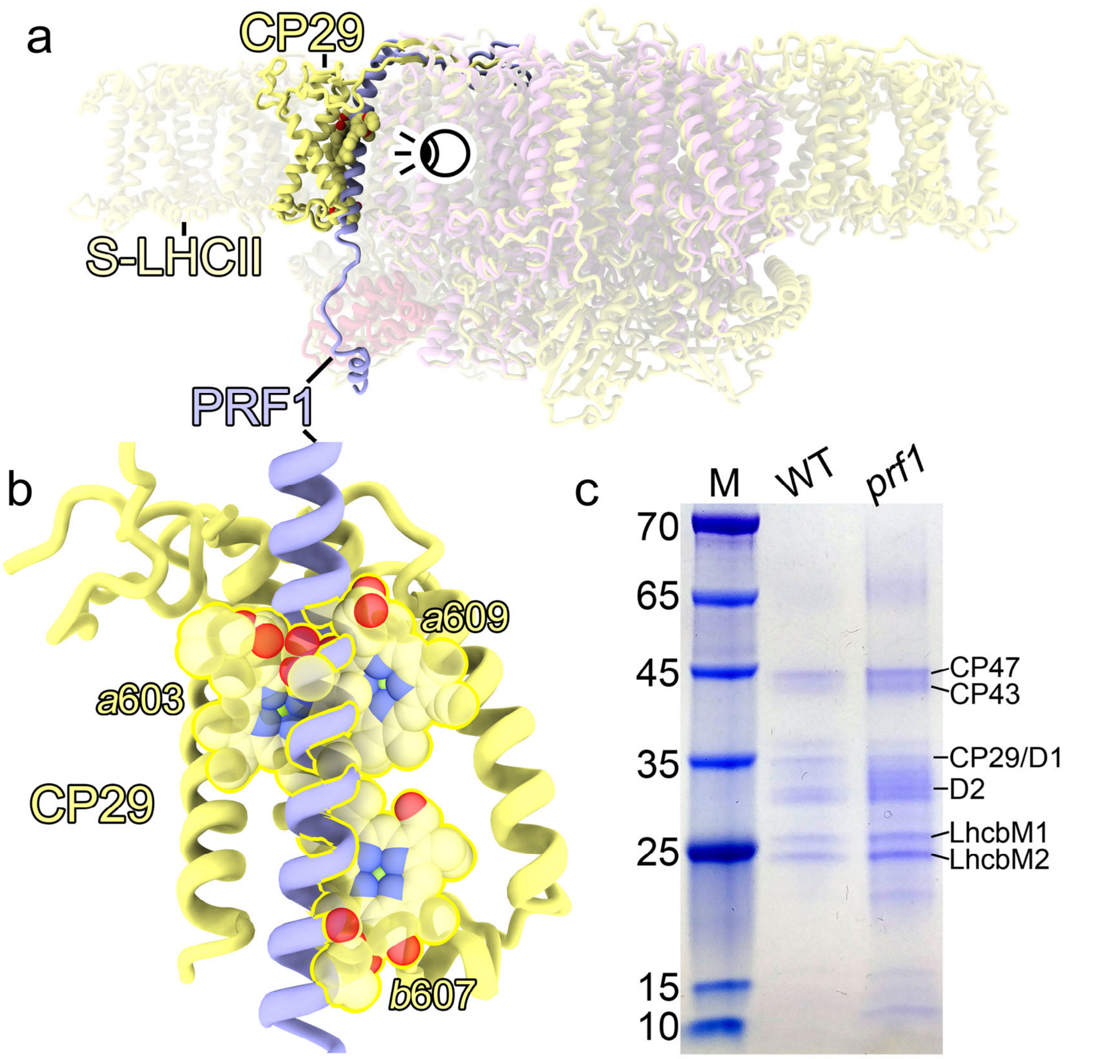

Supplementary Fig. 6: The binding site of PRF1 in the PSII-TPP complex overlaps largely with that of CP29 in the PSII-SC complex. a, Superposition of the PSII-TPP complex (blue, red and magenta) with the low light adapted PSII-SC complex (yellow). The chlorophyll molecules from CP29 which overlap with PRF1 are shown as spheres. The eye symbol represents the viewing angle of (**b**). **b,** A zoom-in view of the local membrane-embedded region showing the overlapping parts of PRF1 and CP29. **c,** SDS-PAGE analysis confirms that the enriched SDG band marked by the light purple arrow in Fig. 4c prepared from the *prf1* mutant cells is composed of PSII-SC complex, whose components are highly similar to those from the corresponding fraction prepared from the WT cells. The experiment was conducted once.

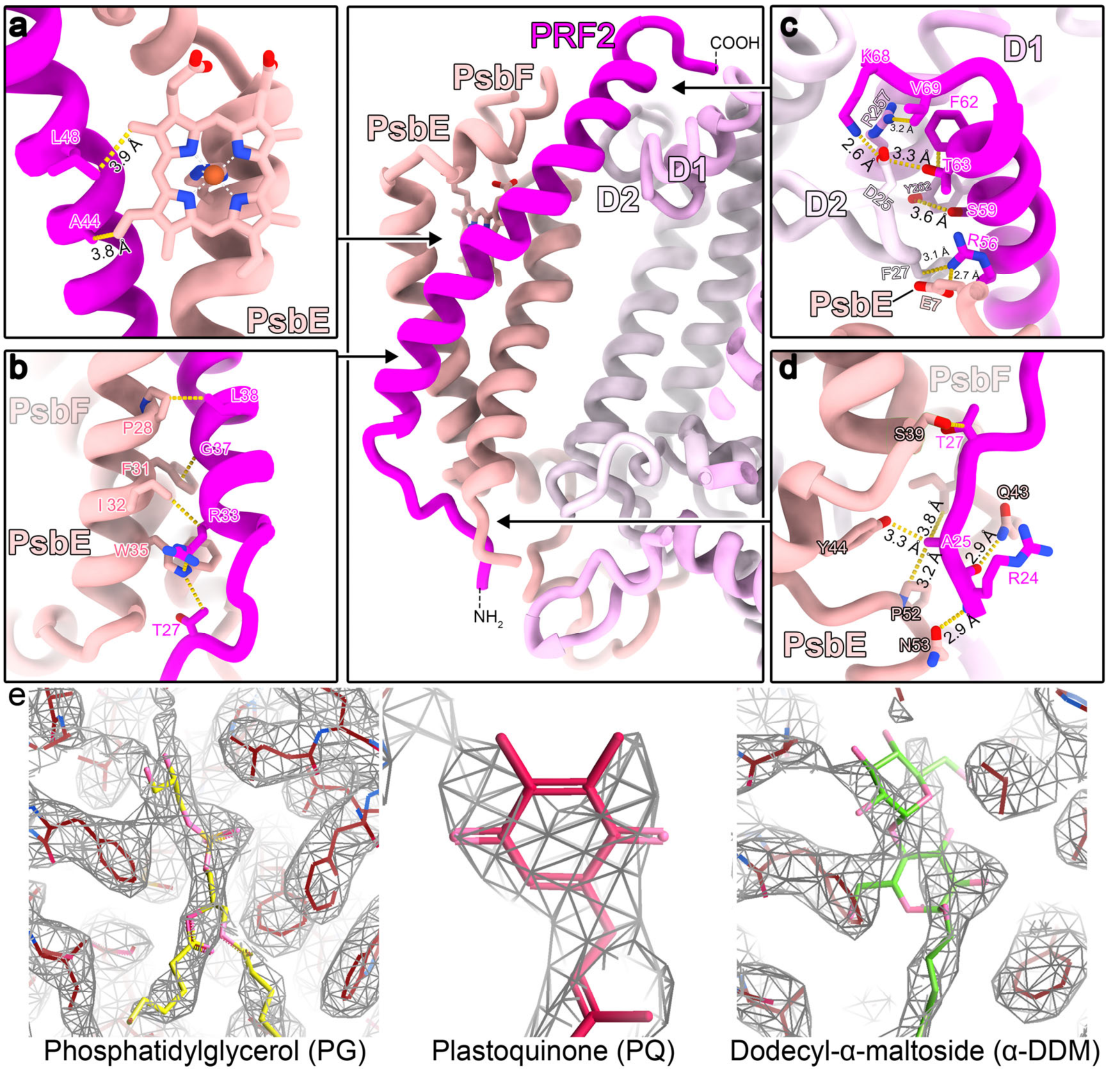

Supplementary Fig. 7: Interactions between PRF2 and PSII-M. a and **b,** The transmembrane helix of PRF2 forms multiple van der Waals interactions with PsbE/F. **c,** The carboxy-terminal region of PRF2 extends to the stromal side and is clamped between PsbE-PsbF and D1-D2. **d,** The amino-terminal region of PRF2 interacts with PsbE/F at the lumenal side. **e,** The local cryo-EM density around the Q_B site with a putative lipid (PG) molecule. The density is overlaid with the structural models of PG (left), PQ (middle) or α-DDM (right). It is evident that the PG model fits well with the density, whereas those of PQ and α-DDM do not match with the density.

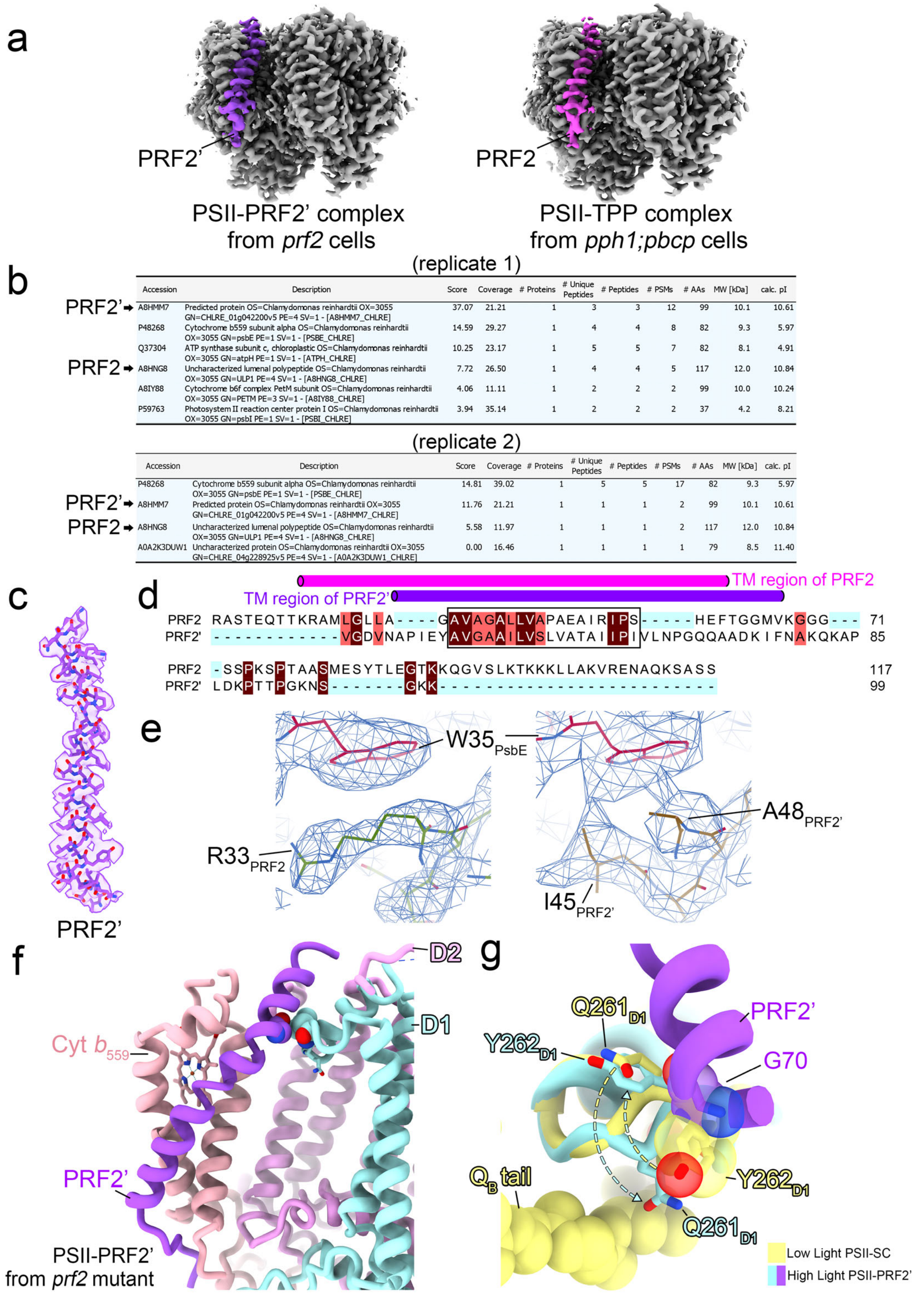

Supplementary Fig. 8: Identification and structural analysis of a newly discovered protein PRF2'. a, The cryo-EM map of the PSII-TPP complex from the *prf2* mutant cells with PRF2' occupying the binding site of PRF2. For comparison, the cryo-EM map of the PSII-TPP complex from the *pph1;pbcp* mutant is shown on the right side. The densities of PRF2' and PRF2 are highlighted in purple and magenta respectively, while the remaining parts are in grey. **b,** Mass spectrometry results from two repeats indicate that the 7-kDa band excised from the SDS-PAGE gel shown in Fig. 1b contains

both PRF2 and PRF2'. The other proteins in the list are likely from contaminants. **c,** Superposition of the PRF2' structural model with the local cryo-EM density map. **d,** Alignment of the amino acid sequences of PRF2 and PRF2'. The transmembrane-helix regions of PRF2 and PRF2' are indicated by the magenta and purple tubes above the sequences, respectively. The highly similar transmembrane regions are framed by the black box. The sequences for the transit peptide regions are omitted for clarity. **e,** The local characteristic cryo-EM densities of PRF2 and PRF2'. The model was superposed with the local cryo-EM density. **f,** Interactions of PRF2' with Cyt b_{559}, D1 and D2. The view is along membrane plane approximately. **g,** Conformational changes at the Q261-Y262 site of the D1 subunit between the low-light PSII-SC and the high-light PSII-PRF2'. The $G70_{PRF2'}$ residue of the high-light PSII-PRF2' complex is in steric hindrance with the $Y262_{D1}$ residue of the low-light PSII-SC. The van der Waals radius of the G70 and Y262 residues are shown as transparent spheres to illustrate their steric hindrance. The dashed arrows indicate the possible directions for the conformational changes of Q261 and Y262 upon binding of PRF2.

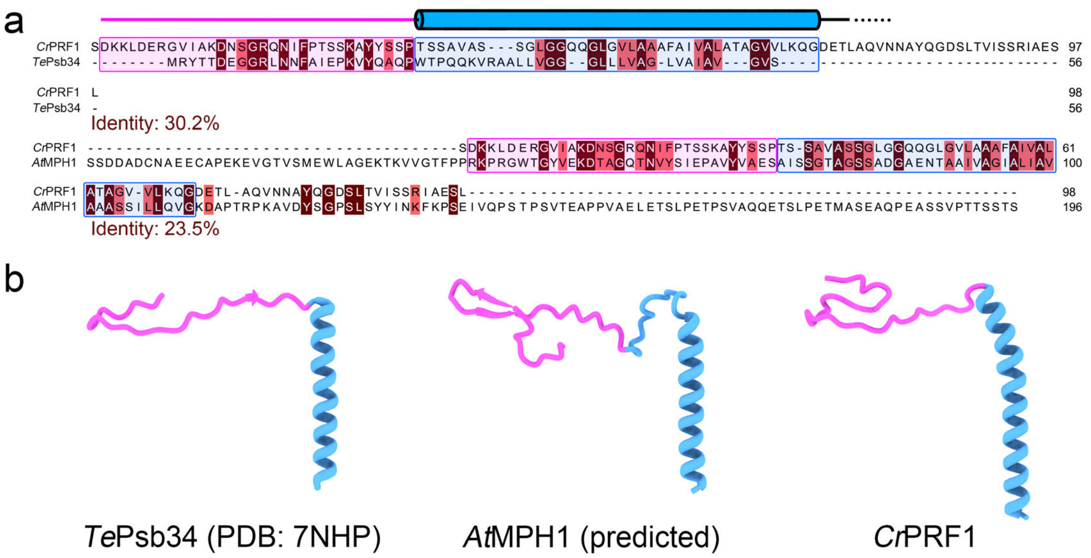

Supplementary Fig. 9: Sequence alignment and structural comparison among *Cr*PRF1, *Te*Psb34 and *At*MPH1. a, Sequence alignment between *Cr*PRF1 and *Te*Psb34, and between *Cr*PRF1 and *At*MPH1. A scheme drawing of the secondary structure of *Cr*PRF1 is labeled above the sequence. The amino-terminal regions and the transmembrane helix regions are framed by magenta and blue rectangles, respectively. The identities of the sequence pairs are listed below the alignment results. **b,** The structural models of *Te*Psb34 (PDB: 7NHP) and *At*MPH1 (predicted by AlphaFold2) in comparison with the cryo-EM structure of *Cr*PRF1 (reported in this work). The carboxy-terminal regions and the disordered amino-terminal region of *At*MPH1 and *Cr*PRF1 are omitted for clarity.

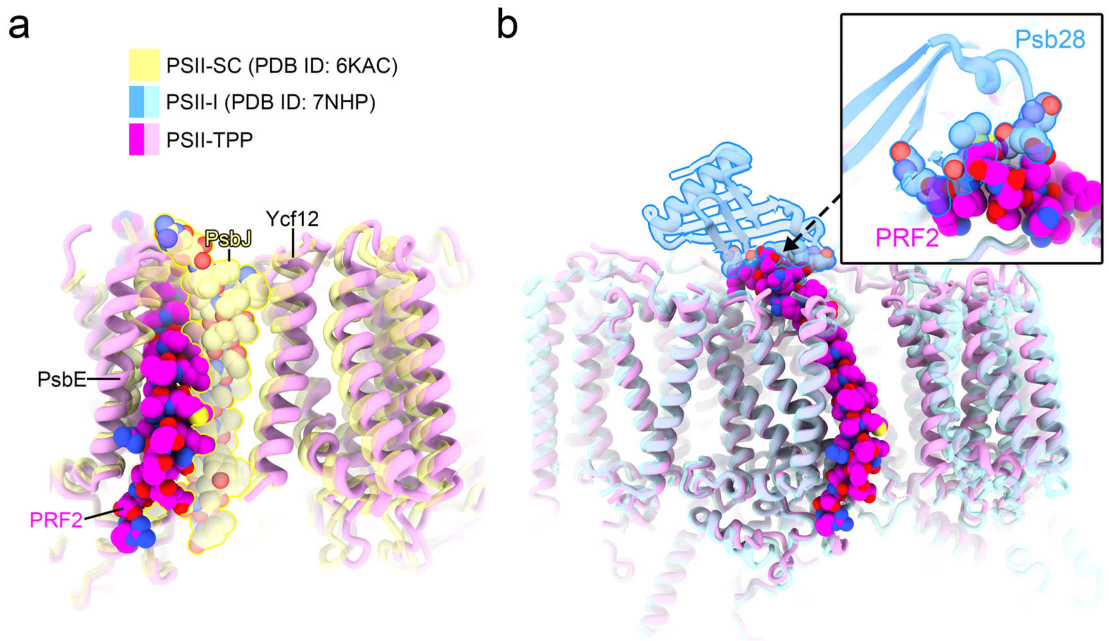

Supplementary Fig. 10: PRF2 partially overlaps with the binding sites of PsbJ and Psb28. a, The structural model of PSII-TPP is superposed with that of the low-light adapted PSII-SC complex (PDB ID: 6KAC). **b**, Superposition of PSII-TPP with the PSII assembly intermediate complex (PSII-I, PDB ID: 7NHP). PsbJ and PRF2 are shown as spheres, Psb28 in PSII-I is shown as cartoon and stick models.

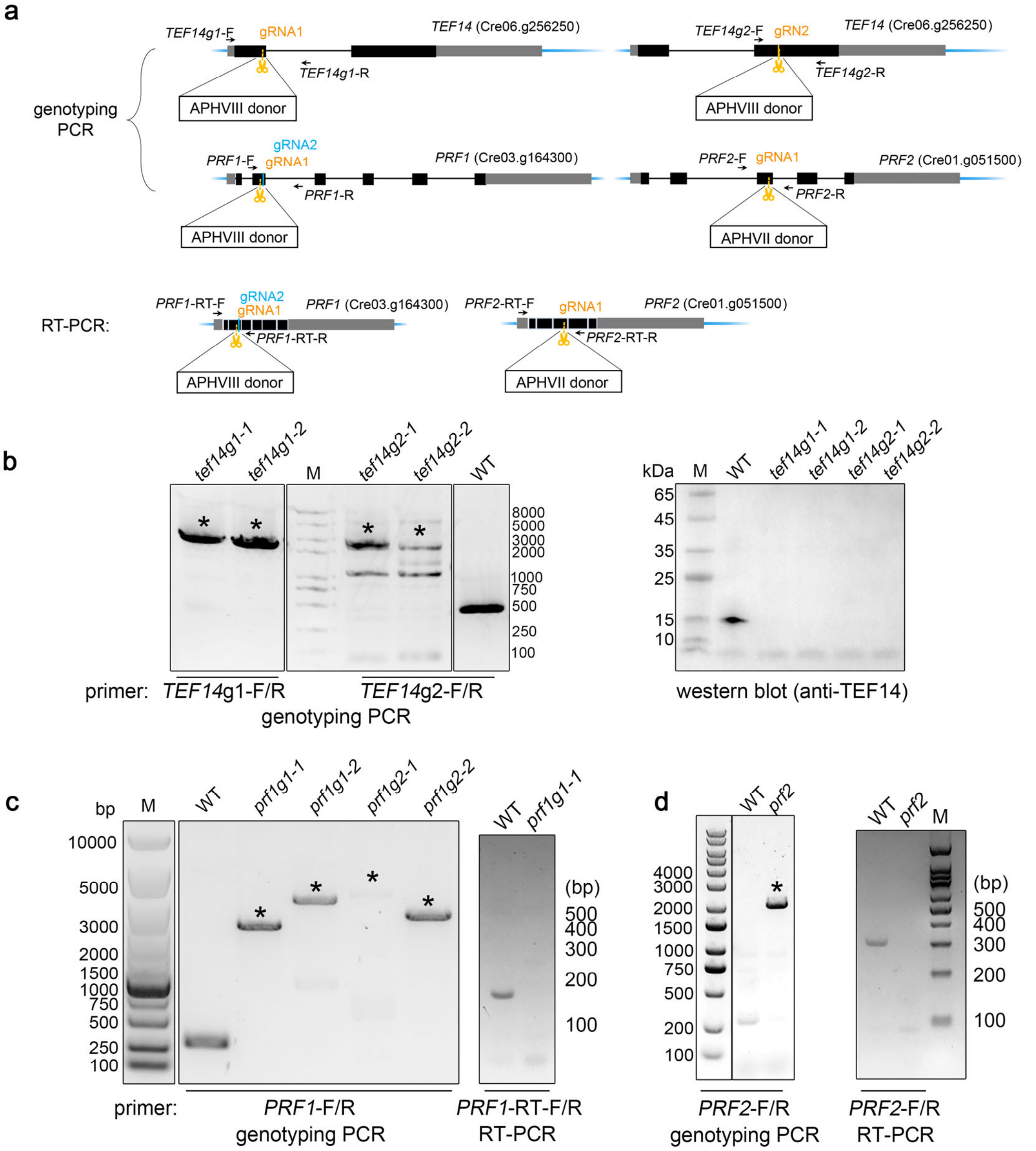

Supplementary Fig. 11: Genotype and western blot validations of the *tef14*, *prf1* and *prf2* mutants. a, A schematic diagram showing the positions of the genotyping PCR primers relative to the *C. reinhardtii* genome and the RT-qPCR primers relative to the cDNA sequences at *TEF14*, *PRF1* and *PRF2* sites. Mutations were achieved by the CRISPR/RNP-based gene editing method to insert the paromomycin- or hygromycin-resistance cassettes into the coding regions of the desired genes. The inserted cassettes are approximately 2.1 kb in length. The PCR primers were designed to be complementary to the *C. reinhardtii* genome or cDNA sequences flanking the insertion sites. **b,** Genotype PCR and western blot analysis for the *tef14* mutants. The antibody against TEF14 was used to verify that mutations were successful at the protein level. **c and d,** Genotype PCR and RT-PCR analysis for the *PRF1* and *PRF2* mutants. For (b), (c) and (d), the PCR bands corresponding to successful insertions with lower migration rates are marked by the asterisks. The genotyping PCR experiments in **b**, **c** and **d** are representatives of two biological replicates with similar results. The experiments for the western blot analysis (b) and RT-qPCR analysis (c and d) were conducted once.

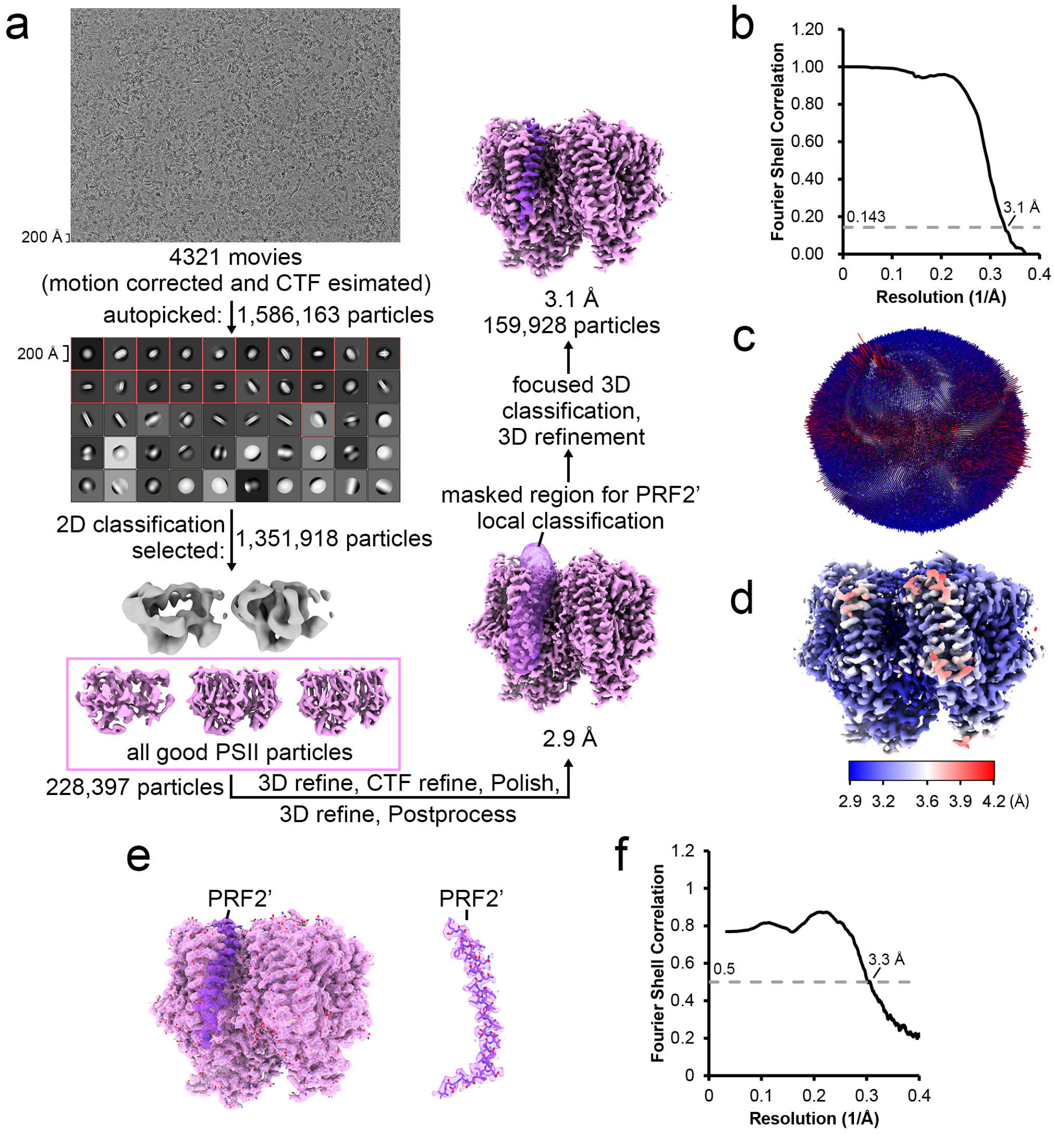

Supplementary Fig. 12: Single particle cryo-EM analysis and evaluation work flow for the PSII-PRF2’ complex from the *C. reinhardtii prf2* mutant strain. a, The overall scheme for the single particle cryo-EM data processing procedure. The micrograph is a representative of 4321 micrographs with similar quality. The selected 2D and 3D classes potentially containing the PSII-M particles are framed in rectangles. The density corresponding to the PRF2’ subunit in the final cryo-EM map is colored in purple, whereas the PSII core complex and the other parts are colored in pink. **b,** The gold standard Fourier shell correlation (GSFSC) curve of the PSII-PRF2’ maps with a resolution-cutoff threshold at 0.143. **c,** Orientational distribution plot of 159,928 3D-refined particles of the PSII-PRF2’ complex yielding a map with well-defined PRF2’ feature at an overall resolution of 3.1 Å. **d,** The local resolution estimations of the final cryo-EM map of the PSII-PRF2’ complex. **e,** The refined structural model of the PSII-PRF2’ complex superposed with the cryo-EM map. **f,** The Fourier shell correlation (FSC) curves between the structural model and the cryo-EM map of the PSII-PRF2’ complex.

Supplementary Table 1: Cryo-EM data collection, model refinement and validation statistics.

**Data collection and processing**	**PSII-TPP complex**	**PSII-PRF2' complex**
Magnification	130,000	22,500
Voltage (kV)	300	300
Electron exposure (e^{-}/Å^2)	60	60
Defocus range (μm)	-1 to -1.5	-1 to -1.5
Pixel size (Å)	1.04	1.35
Symmetry imposed	*C*1	*C*1
Initial particle images (no.)	4,333,556	1,586,163
Final particle images (no.)	510,932	159,928
Map resolution (Å)	2.6	2.9
FSC threshold	0.143	0.143
Map resolution range (Å)	2.5-7.0	2.9-6.4
**Model refinement**		
Initial model used (PDB code)	6KAC	8KDE
Model resolution (Å)	3.1	3.2
FSC threshold	0.5	0.5
**Model composition**		
Non-hydrogen atoms	22,087	20,267
Protein residues	2,344	2,101
Ligands	72	71
Map sharpening B factor (Å^2)	-71.73	-70
**B factors (Å^2)**		
Protein	70.42	52.03
Ligands	63.84	49.86
**R.m.s. deviations**		
Bond lengths (Å)	0.003	0.003
Bond angles (°)	0.714	0.682
**Validation**		
MolProbity score	1.51	1.58
Clashscore	4.56	7.99
Poor rotamers (%)	0	0
**Ramachandran plot**		
Favored (%)	96	97.19
Allowed (%)	3.82	2.71
Disallowed (%)	0.17	0.10

Supplementary Table 2: Sequences of the single guide RNAs for CRISPR/RNP-based gene editing.

sgRNA	Sequence
PRF1-gRNA1	mC*mU*mU*ACUUCUUGUCCGAAGCGGUUUUAGAGCUAGAAAUAGCAAGUUAAAAUAAGGCUAGUCCGUUAUCAACUUGAAAAAGUGGCACCGAGUCGGUGCU*mU*mU*mU
PRF1-gRNA2	mU*mU*mC*GGACAAGAAGUAAGCAAGUUUUAGAGCUAGAAAUAGCAAGUUAAAAUAAGGCUAGUCCGUUAUCAACUUGAAAAAGUGGCACCGAGUCGGUGCU*mU*mU*mU
PRF2-gRNA1	mA*mU*mC*CCCUCGCACGAGUUCACGUUUUAGAGCUAGAAAUAGCAAGUUAAAAUAAGGCUAGUCCGUUAUCAACUUGAAAAAGUGGCACCGAGUCGGUGCU*mU*mU*mU
TEF14-gRNA1	mC*mU*mU*GGGGGCAUACAUCAGCGGUUUUAGAGCUAGAAAUAGCAAGUUAAAAUAAGGCUAGUCCGUUAUCAACUUGAAAAAGUGGCACCGAGUCGGUGCU*mU*mU*mU
TEF14-gRNA2	mG*mC*mU*CUUGGCCAGCUUGUACAGUUUUAGAGCUAGAAAUAGCAAGUUAAAAUAAGGCUAGUCCGUUAUCAACUUGAAAAAGUGGCACCGAGUCGGUGCU*mU*mU*mU

Note:
m = 2'-O-methyl modification
* = phosphorothioate modification
The scaffold sequences of sgRNAs are underlined.

Supplementary Table 3: Sequences of the primers used in donor DNA amplification for CRISPR/RNP-based gene editing.

sgRNA	Donor DNA amplification primer sequence
TEF14g1-F	GGCGACCCCCAACACCGCCTCGCGTCGTGCTATTCTGGGCGTGGCGCTGCTGCACTAGTCACACGAGCCC
TEF14g1-R	GGCGACCCGCAGCCACACAGCTCCGCAAGCGACTCACCAGGAATCAGGGCCCACTCTGGGTCTCTAGCGC
TEF14g2-F	CGAGAACCTGACCGACCGCCGCCTGGAGCAGGAGCTGGTGCCGGTGCAGATGCACTAGTCACACGAGCCC
TEF14g2-R	GACTCGGACAGGGTGCTGGCCGCGCCCTTCAGGTCGCCGCTCTCCAGCTGCCACTCTGGGTCTCTAGCGC
PRF1g1-F	ACGTCCAGCGCTGCTGTAGCGCCCGCGCGGCCAGCTCGCGTGAGCCATATTGCACTAGTCACACGAGCCC
PRF1g1-R	CTAGTGACATGCGAGCGAATCAGAACGTATCCGTATGTTTCTTGGCGGTACCACTCTGGGTCTCTAGCGC
PRF1g2-F	TCCAGCGCTGCTGTAGCGCCCGCGCGGCCAGCTCGCGTGAGCCATATCGTTGCACTAGTCACACGAGCCC
PRF1g2-R	TGACTAGTGACATGCGAGCGAATCAGAACGTATCCGTATGTTTCTTGGCGCCACTCTGGGTCTCTAGCGC
PRF2g1-F	TTGCTGCCGCAGCCGTCGCTGGTGCCCTGCTGGTGGCTCCCGCTGAGGCCGATATCAAGCTTCTTTCTTGCGC
PRF2g1-R	AATTCTTGCGTCTTACCACTTGCGCAAAACGCAACTTACCCTTCACCATACCAAGCTTCCATGGGATGAC

Supplementary Table 4: Sequences of the primers used for genotype verification of the potential mutants. The two gRNA sites for *PRF1* can share the same genotyping primers.

sgRNA	Genotyping primer sequences
TEF14g1-F	CCTTCCCAATCCACCACAC
TEF14g1-R	CTCAGCAAATGTAGCAGCTG
TEF14g2-F	CGAGGAGGATGAGGACCTG
TEF14g2-R	GTCCGACACCAGGCTCAC
PRF1-F	GTTGCATTCCTCGTAGGTCC
PRF1-R	AGCCACGCGAGTTCAGATC
PRF2g1-F	GCTGATCCCGGTCTGTGC
PRF2g1-R	GAGCGTAACGCGCATGAAAC

Supplementary Table 5: Sequences of the primers used for quantitative reverse transcription polymerase chain reaction (RT-qPCR).

Gene	Forward Primers (5’ to 3’)	Reverse Primers (5’ to 3’)
TEF14	TCGCGTCGTGCTATTCTGG	CCTCATCCTCCTCGTCAGGAA
PRF1	CGCTTCGGACAAGAAGCTC	TGCTGGTGGGGAAGATGTTTT
PRF2	GAAGAAGCTGCTGGCCAAGG	CAGCAAACGTGGAACTCGTG
CBLP	CAAGTACACCATTGGCGAGC	CTTGCAGTTGGTCAGGTTCC

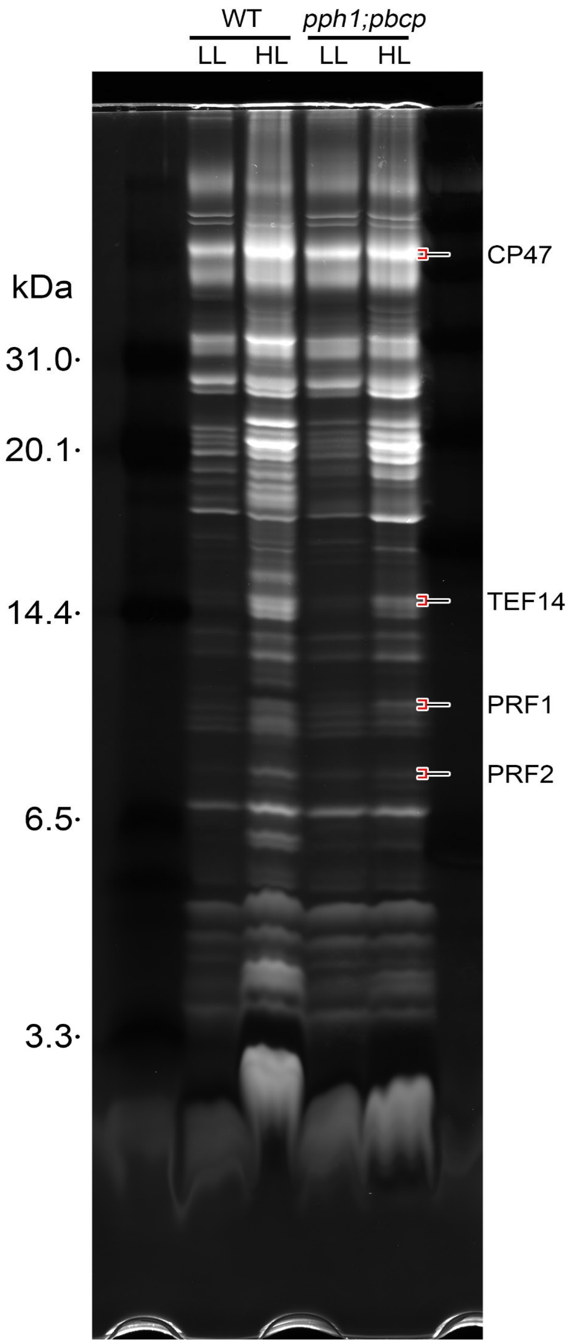

238 **Source Data for Supplementary Fig. 1a.**

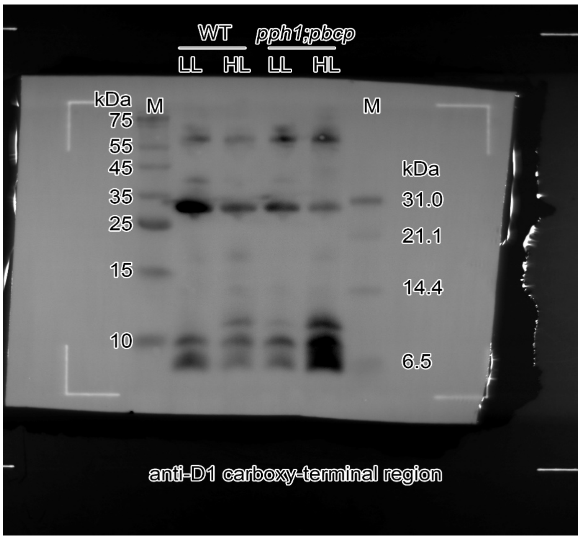

Source Data for Supplementary Fig. 3c.

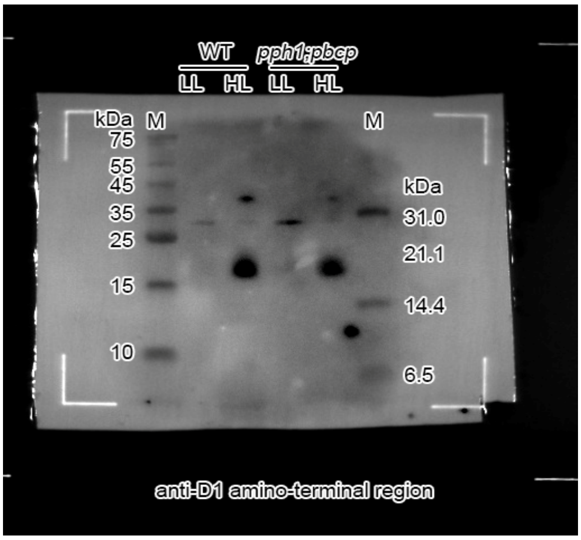

Source Data for Supplementary Fig. 3d.

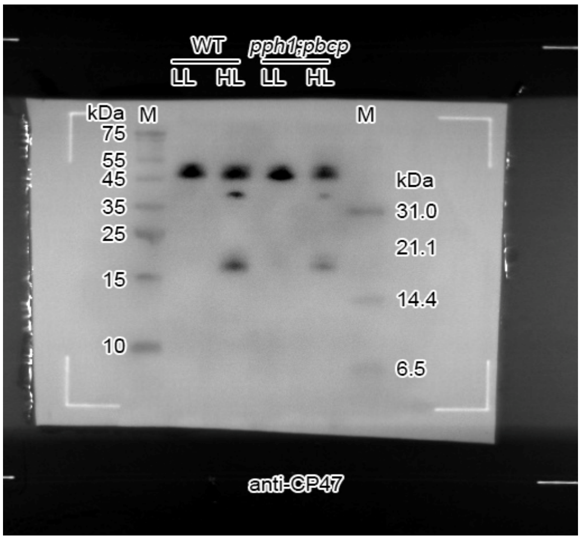

Source Data for Supplementary Fig. 3e.

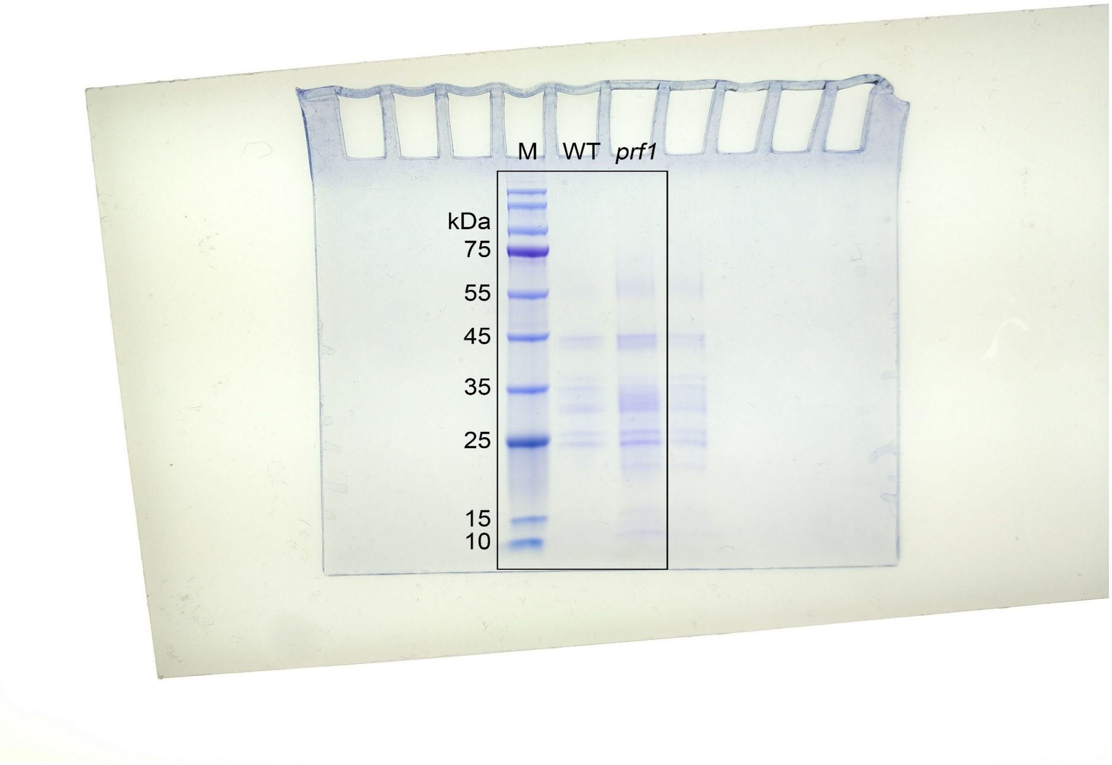

Source Data for Supplementary Fig. 6c.

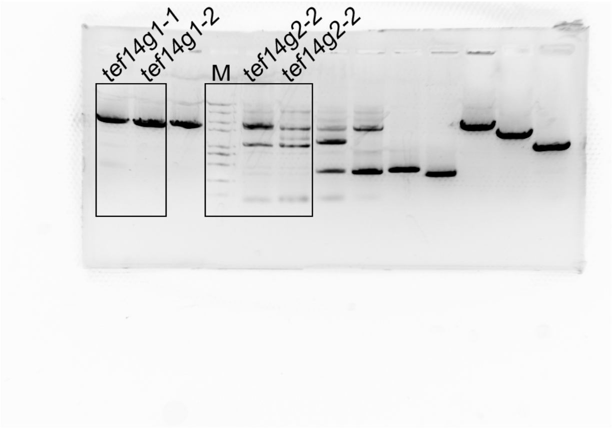

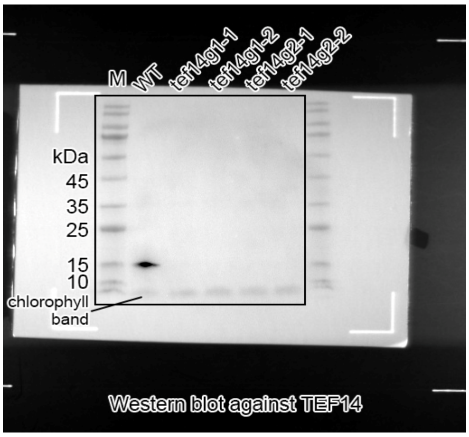

Source Data for Supplementary Fig. 11b. Left panel: The genotype PCR results for WT and the *tef14* mutants; Right panel: The western blot analysis for WT and the *tef14* mutants using the antibody against TEF14.

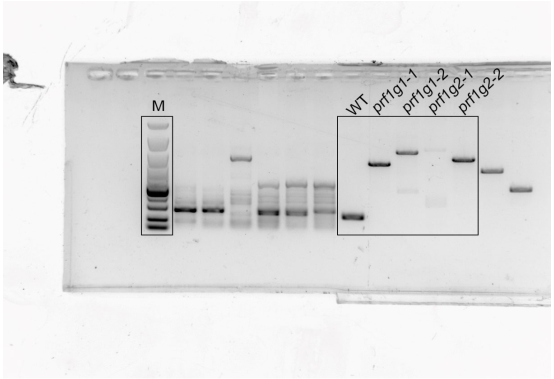

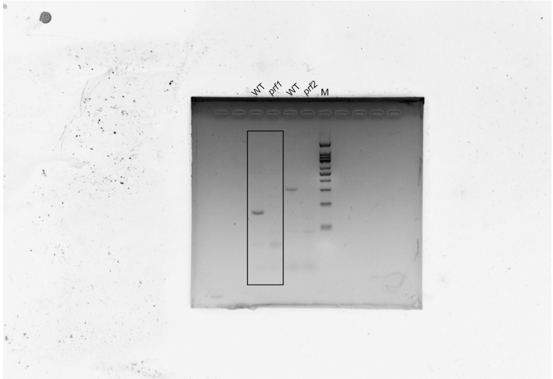

Source Data for Supplementary Fig. 11c. Left panel: The genotype PCR result for WT and the *prf1* mutants; Right panel: The quantitative reverse transcription polymerase chain reaction (RT-qPCR) analysis for WT and the *prf1* mutants.

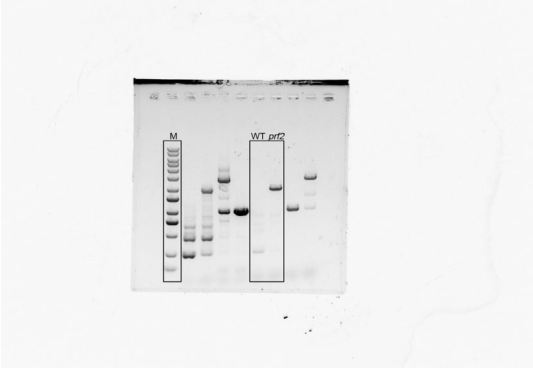

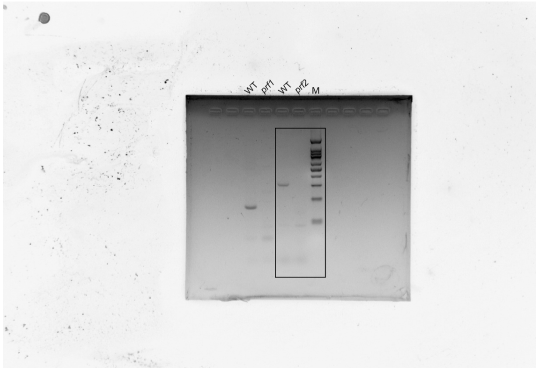

Source Data for Supplementary Fig. 11d. Left panel: The genotype PCR result for WT and the *prf2* mutant; Right panel: The RT-qPCR analysis for WT and the *prf2* mutant.
