# Supplementary material for: Structural basis for an early stage of the photosystem II repair cycle in Chlamydomonas reinhardtii
Source: Nat Commun. 2024 Jun 18;15:5211. doi: 10.1038/s41467-024-49532-2 (PMC11189392; doi:10.1038/s41467-024-49532-2)
Supplement: Supplementary file 2 — Reporting Summary [file 41467_2024_49532_MOESM2_ESM.pdf]

nature portfolio

Corresponding author(s): Zhenfeng Liu and Xiaobo Li

Last updated by author(s): Jun 4, 2024

Reporting Summary

Nature Portfolio wishes to improve the reproducibility of the work that we publish. This form provides structure for consistency and transparency in reporting. For further information on Nature Portfolio policies, see our Editorial Policies and the Editorial Policy Checklist.

Statistics

For all statistical analyses, confirm that the following items are present in the figure legend, table legend, main text, or Methods section.

n/a	Confirmed	
☐	☒	The exact sample size (n) for each experimental group/condition, given as a discrete number and unit of measurement
☐	☒	A statement on whether measurements were taken from distinct samples or whether the same sample was measured repeatedly
☐	☒	The statistical test(s) used AND whether they are one- or two-sided<br>*Only common tests should be described solely by name; describe more complex techniques in the Methods section.*
☒	☐	A description of all covariates tested
☒	☐	A description of any assumptions or corrections, such as tests of normality and adjustment for multiple comparisons
☐	☒	A full description of the statistical parameters including central tendency (e.g. means) or other basic estimates (e.g. regression coefficient) AND variation (e.g. standard deviation) or associated estimates of uncertainty (e.g. confidence intervals)
☐	☒	For null hypothesis testing, the test statistic (e.g. F, t, r) with confidence intervals, effect sizes, degrees of freedom and P value noted<br>*Give P values as exact values whenever suitable.*
☒	☐	For Bayesian analysis, information on the choice of priors and Markov chain Monte Carlo settings
☒	☐	For hierarchical and complex designs, identification of the appropriate level for tests and full reporting of outcomes
☒	☐	Estimates of effect sizes (e.g. Cohen's d, Pearson's r), indicating how they were calculated

Our web collection on statistics for biologists contains articles on many of the points above.

Software and code

Policy information about availability of computer code

Data collection	SerialEM v.3.6, FlexControl v.3.4
Data analysis	RELION v.4.0, UCSF Chimera v.1.14 and ChimeraX v.1.5, WinCoot v.0.9.8, PHENIX v.1.19, Peakview v.2.1, MASCOT 2022, Origin 2021, ImageJ 1.53q5, CCP4i2 v.1.1.0, CryoEF, Graphpad Prism 10, ChemDraw 20.0

For manuscripts utilizing custom algorithms or software that are central to the research but not yet described in published literature, software must be made available to editors and reviewers. We strongly encourage code deposition in a community repository (e.g. GitHub). See the Nature Portfolio guidelines for submitting code & software for further information.

Data

Policy information about availability of data

All manuscripts must include a data availability statement. This statement should provide the following information, where applicable:

- Accession codes, unique identifiers, or web links for publicly available datasets
- A description of any restrictions on data availability
- For clinical datasets or third party data, please ensure that the statement adheres to our policy

The previously published structural models of the Thermostichus vulcanus PSII complex, the Chlamydomonas reinhardtii PSII-LHCII C2S2 type supercomplex and the Thermosynechococcus vestitus BP-1 PSII-I assembly intermediate complex can be accessed from the Protein Data Band under the accession codes 3WU2 [https://doi.org/10.2210/pdb3WU2/pdb], 6KAC [https://doi.org/10.2210/pdb6KAC/pdb] and 7NHP [https://doi.org/10.2210/pdb7NHP/pdb], respectively. The composite cryo-EM map of the Chlamydomonas reinhardtii PSII-TPP complex and its corresponding atomic coordinates have been deposited in the Electron Microscopy Data

Bank and the Protein Data Bank under the accession codes EMD-37133 [https://www.ebi.ac.uk/emdb/EMD-37133] and 8KDE [https://www.rcsb.org/structure/unreleased/8KDE] respectively. The cryo-EM map of the Chlamydomonas reinhardtii PSII-PRF2' complex and its corresponding atomic coordinates have been deposited in the Electron Microscopy Data Bank and the Protein Data Bank under the accession codes EMD-60026 [https://www.ebi.ac.uk/emdb/EMD-60026] and 8ZEE [https://www.rcsb.org/structure/unreleased/8ZEE] respectively. The mass spectrometry proteomics data generated in this study have been deposited to the ProteomeXchange Consortium via the PRIDE partner repository with the dataset identifier PXD052618 [https://www.ebi.ac.uk/pride/archive/projects/PXD052618]. All data analyzed during this study are included in this Article and its Supplementary Information. Source data are provided with this paper. The tef14, prf1 and prf2 mutant strains of Chlamydomonas reinhardtii generated in this work and other related materials are available from the corresponding authors upon request.

Research involving human participants, their data, or biological material

Policy information about studies with human participants or human data. See also policy information about sex, gender (identity/presentation), and sexual orientation and race, ethnicity and racism.

Reporting on sex and gender	N/A
Reporting on race, ethnicity, or other socially relevant groupings	N/A
Population characteristics	N/A
Recruitment	N/A
Ethics oversight	N/A

Note that full information on the approval of the study protocol must also be provided in the manuscript.

Field-specific reporting

Please select the one below that is the best fit for your research. If you are not sure, read the appropriate sections before making your selection.

[x] Life sciences [ ] Behavioural & social sciences [ ] Ecological, evolutionary & environmental sciences

For a reference copy of the document with all sections, see nature.com/documents/nr-reporting-summary-flat.pdf

Life sciences study design

All studies must disclose on these points even when the disclosure is negative.

Sample size	No statistical methods were used to predetermine the sample size. For biochemical experiments, most of the experiments were performed with at least 3 technical replicates and at least 2 biological replicates. This approach is supported by the common practices outlined in previous biochemical research, which suggest that such sample sizes are adequate for detecting significant differences in most biochemical assays. For cryo-EM analysis, the sample size of 5,189 micrographs and 4,321 micrographs were sufficient for obtaining the cryo-EM map with resolutions of 2.6 Å and 2.9 Å for the PSII-TPP complex and PSII-PRF2' complex, respectively.
Data exclusions	The initial cryo-EM images were screened manually to exclude those with low contrast, thick ice or severe ice contaminations, which is a standard procedure for cryo-EM data analysis. No biochemical data have been excluded.
Replication	Two cryo-EM datasets were collected from one cryo-EM grid sample and combined for further data processing. The biochemical experiments were repeated at least three times, and were all successfully reproduced. The number of independent experiments and replicates are indicated in the related figure legends.
Randomization	For biochemical analysis, the samples were all mixed evenly before sampling to ensure randomness during sample allocation. For the cryo-EM work, the particles were randomly distributed into two halves, and were reconstructed separately to generate two half-maps. The Fourier shell correlation were then calculated by using the two half-maps to estimate the quality of reconstruction.
Blinding	Blinding is not relevant for the structural analysis in our work as no allocation into experimental groups was needed. The investigators need to select the target particles with the PSII-repair factors for reconstruction and further analysis during the data processing procedure.

Reporting for specific materials, systems and methods

We require information from authors about some types of materials, experimental systems and methods used in many studies. Here, indicate whether each material, system or method listed is relevant to your study. If you are not sure if a list item applies to your research, read the appropriate section before selecting a response.

Materials & experimental systems

n/a	Involved in the study
☐	☒ Antibodies
☒	☐ Eukaryotic cell lines
☒	☐ Palaeontology and archaeology
☒	☐ Animals and other organisms
☒	☐ Clinical data
☒	☐ Dual use research of concern
☐	☒ Plants

Methods

n/a	Involved in the study
☒	☐ ChIP-seq
☒	☐ Flow cytometry
☒	☐ MRI-based neuroimaging

Antibodies

Antibodies used	Antibodies against the carboxy-terminal fragment of D1 (AS05084, used at a dilution of 1:10,000) and the CP47 apoprotein (AS04038, used at a dilution of 1:1,000) were purchased from Agrisera. Antibodies against the amino-terminal of D1 (PHY0057, used at a dilution of 1:1,000) and the PsbO subunit (PHY0094A, used at a dilution of 1:1,000) were purchased from PhytoAB. Multi-clonal antibody against the TEF14 subunit was prepared by ABclonal (Wuhan, China) using the purified recombinant CrTEF14 protein expressed in E. coli. The antibody against TEF14 was used at a dilution of 1:1,000.
Validation	The commercial antibodies were proved to be reactive according to the manufacturer's manual and our own experiments. The TEF14 antibody was proved to be effective and specific during our western blot analysis.

Dual use research of concern

Policy information about dual use research of concern

Hazards

Could the accidental, deliberate or reckless misuse of agents or technologies generated in the work, or the application of information presented in the manuscript, pose a threat to:

No	Yes
☒	☐ Public health
☒	☐ National security
☒	☐ Crops and/or livestock
☒	☐ Ecosystems
☒	☐ Any other significant area

Experiments of concern

Does the work involve any of these experiments of concern:

No	Yes
☒	☐ Demonstrate how to render a vaccine ineffective
☒	☐ Confer resistance to therapeutically useful antibiotics or antiviral agents
☒	☐ Enhance the virulence of a pathogen or render a nonpathogen virulent
☒	☐ Increase transmissibility of a pathogen
☒	☐ Alter the host range of a pathogen
☒	☐ Enable evasion of diagnostic/detection modalities
☒	☐ Enable the weaponization of a biological agent or toxin
☒	☐ Any other potentially harmful combination of experiments and agents
