# Supplementary material for: Structural basis for an early stage of the photosystem II repair cycle in Chlamydomonas reinhardtii
Source: Nat Commun. 2024 Jun 18;15:5211. doi: 10.1038/s41467-024-49532-2 (PMC11189392; doi:10.1038/s41467-024-49532-2)
Supplement: Supplementary file 3 — Description of Additional Supplementary Files [file 41467_2024_49532_MOESM3_ESM.pdf]

Supplementary Movie 1:

Mobility and conformational changes of TEF14 and PRF1 associated with a damaged PSII core complex. The transparent cryo-EM maps superposed with the model were shown in some of the frames in the movie. It is a morph movie generated with the structural models fitted in the corresponding cryo-EM maps of two different 3D classes with TEF14 trapped in distinct conformations.
